# Selective Sampling of Species and Fossils Influences Age Estimates Under the Fossilized Birth–Death Model

**DOI:** 10.3389/fgene.2019.01064

**Published:** 2019-10-31

**Authors:** Michael Matschiner

**Affiliations:** ^1^Department of Palaentology and Museum, University of Zurich, Zurich, Switzerland; ^2^Centre of Ecological and Evolutionary Synthesis, Department of Biosciences, University of Oslo, Oslo, Norway

**Keywords:** phylogeny, bayesian inference, divergence-time estimation, fossil, diversified sampling, BEAST 2, fossilized birth–death, CladeAge

## Abstract

The fossilized birth–death (FBD) model allows the estimation of species divergence times from molecular and fossil information in a coherent framework of diversification and fossil sampling. Some assumptions of the FBD model, however, are difficult to meet in phylogenetic analyses of highly diverse groups. Here, I use simulations to assess the impact of extreme model violations, including diversified sampling of species and the exclusive use of the oldest fossils per clade, on divergence times estimated with the FBD model. My results demonstrate that selective sampling of fossils can produce dramatically overestimated divergence times when the FBD model is used for inference, due to an interplay of underestimates for the model parameters net diversification rate, turnover, and fossil-sampling proportion. In contrast, divergence times estimated with CladeAge, a method that uses information about the oldest fossils per clade together with estimates of sampling and diversification rates, are accurate under these conditions. Practitioners of Bayesian divergence-time estimation should therefore ensure that the dataset conforms to the expectations of the FBD model, or estimates of sampling and diversification rates should be obtained *a priori* so that CladeAge can be used for the inference.

## Introduction

With increases in the sizes of molecular datasets and improvements to inference methodology, our understanding of the timeline of evolution has grown tremendously over the past two decades. One of the most significant methodological developments for the estimation of divergence times has been the fossilized birth–death (FBD) model (Stadler, 2010; Heath et al., 2014), a phylogenetic framework that combines the two processes of species diversification and fossil sampling. By using fossils as tips or sampled ancestors in the phylogeny, the FBD model is able to estimate the probability that species fossilize before their extinction and it accounts for this probability in the inference. The FBD model thus overcomes a limitation of the commonly applied “node dating” approach in which only the oldest fossils of some clades are used to define constraints on the divergence times among these clades: As fossils can provide reliable evidence for the minimum age of a clade but are only vaguely informative about maximum ages when the sampling process is not included in the model (Benton and Donoghue, 2007), the placement of maximum ages in node dating is often controversial, even though it is essential for the inference (Marjanović and Laurin, 2007; Warnock et al., 2012). Because age constraints with minimum and maximum ages are not required with the FBD model, estimates obtained with this model do not depend on the controversial specification of those constraints and may thus be generally more reliable.

The FBD model was first available for inference in the program DPPDIV (Heath et al., 2014), allowing the estimation of divergence times from a molecular dataset and a user-provided tree with a fixed topology. The dependence on a known topology has been relaxed in subsequent implementations of the model in BEAST 2 (Gavryushkina et al., 2014; Gavryushkina et al., 2017; Bouckaert et al., 2019), MrBayes (Ronquist et al., 2012; Zhang et al., 2016), and RevBayes (Höhna et al., 2016) all of which also allow the inference of fossil positions based on morphological information instead of requiring the user to know their positions *a priori*. The FBD model has further matured with the integration of stratigraphic-range information and different speciation modes (Silvestro et al., 2018; Stadler et al., 2018), time-variable diversification and sampling (Gavryushkina et al., 2014), coalescent processes (Ogilvie et al., 2018), and the estimation of divergence times without assuming molecular or morphological clocks (Didier and Laurin, 2018).

The accuracy of age estimates obtained with the FBD model has been tested with simulations in multiple studies that all confirmed reliable inference (Gavryushkina et al., 2014; Heath et al., 2014; Zhang et al., 2016; Matschiner et al., 2017). The simulations in these studies mostly did not violate the assumptions of the FBD model, which include that either all simulated species and fossils or a randomly selected subset of these are used for the inference. A “complete sampling” scheme (or at least nearly complete sampling) for species and fossils was also applied in a number of empirical studies using the FBD, to estimate divergence times among, e.g., bears (Heath et al., 2014), penguins (Gavryushkina et al., 2017), and beech trees (Renner et al., 2016); however, most empirical datasets may not be completely sampled. Instead, extant species may often be missing from phylogenetic datasets, for example due to limited availability of sequence data. The incompleteness of taxon sets is usually amplified in phylogenetic analyses of larger clades, where the inclusion of all species would be computationally infeasible or the generation of molecular data for all species would be too costly. Moreover, the selection of species for such analyses may rarely be uniformly random and instead “diversified” sampling of extant species may be more common (Höhna et al., 2011; Höhna, 2014), because researchers often aim to include representatives of each major group within the studied clade (e.g. Meredith et al., 2011; Jarvis et al., 2014; dos Reis et al., 2015; Barba-Montoya et al., 2018; Musilova et al., 2019).

Like the sampling of extant species in empirical analyses, the inclusion of fossils may also often be neither complete nor random. For larger clades with high preservation potential, complete sampling of fossils may not be possible due to their sheer numbers, and instead of applying random sampling of fossils as an alternative, researchers may want to ensure that the earliest records are included in the dataset. Thus, both the sampling of extant species and of fossils is probably selective in most empirical datasets used in analyses with the FBD model; however, the degree to which the FBD model is robust to these model violations has so far not been tested with simulations.

As another alternative to node dating, Matschiner et al. (2017) developed CladeAge, an approach that estimates divergence times based on information about the oldest fossils of clades, in combination with estimates of sampling and diversification rates. Specifically, CladeAge uses this information to derive probability distributions for the ages of individual clades under a model of time-homogeneous diversification and fossil sampling, and those probability distributions are then used as calibration densities in phylogenetic divergence-time estimation. By assuming Poisson processes for diversification and fossil sampling, the derivation of probability distributions in CladeAge is essentially based on the FBD model, but the processes are truncated at the first sampling event. As the term “FBD model” is commonly understood to describe the process continued to the present, I will use this term as a synonym for the implementations of this untruncated model (e.g., in BEAST 2, MrBayes, or RevBayes), and I will use the term “CladeAge” to refer to the combined approach of model-based quantification of calibration densities per clade and the use of these densities for divergence-time estimation. Like the FBD model, the performance of CladeAge has been tested with simulations that confirmed reliable inference (Matschiner et al., 2017); however, as in the case of the FBD model, these simulations matched the expectations of the method in terms of sampling of extant species and fossils.

Here, I test the performance of both the FBD model and CladeAge in scenarios of model violations that include a strict diversified sampling scheme for extant species and the exclusive use of the oldest fossils per clade (the latter only violates the FBD model but matches the assumptions of CladeAge). While these scenarios are probably more extreme than the model violation in almost all empirical analyses, I expect that the results will provide valuable clues about the robustness of the inference with the two approaches.

## Method

### Simulations

I used forward simulation to generate phylogenetic trees as in Matschiner et al. (2017), with branch lengths corresponding to time. In these simulations, I set the age of the first divergence to 100 time units in the past and applied a constant-rate birth–death process (Gernhard, 2008) with cladogenetic speciation (hereafter: “speciation”) rate λ = 0.12 and extinction rate μ = 0.06 to generate the tree. The net diversification rate (λ−μ) was thus 0.06 and the turnover (λ/μ) was 0.5, and these rates applied to all branches of the tree. I repeated this simulation until 20 trees were found that had between 4,000 and 5,000 extant species, and I discarded all trees that did not fulfill this condition. Age and species richness of the simulated phylogenies were thus roughly comparable to those of placental mammals (Meredith et al., 2011; Stadler, 2011) if we assume one time unit to correspond to one million years. I added a simulated fossil record to all branches of the trees, assuming a homogeneous Poisson process of fossil sampling with sampling rate ψ = 0.01 and thus a fossil-sampling proportion of ψ/(μ+ψ) = 0.143 (Gavryushkina et al., 2014). The recorded fossil ages were assumed to be known without error.

**Figure 1:**
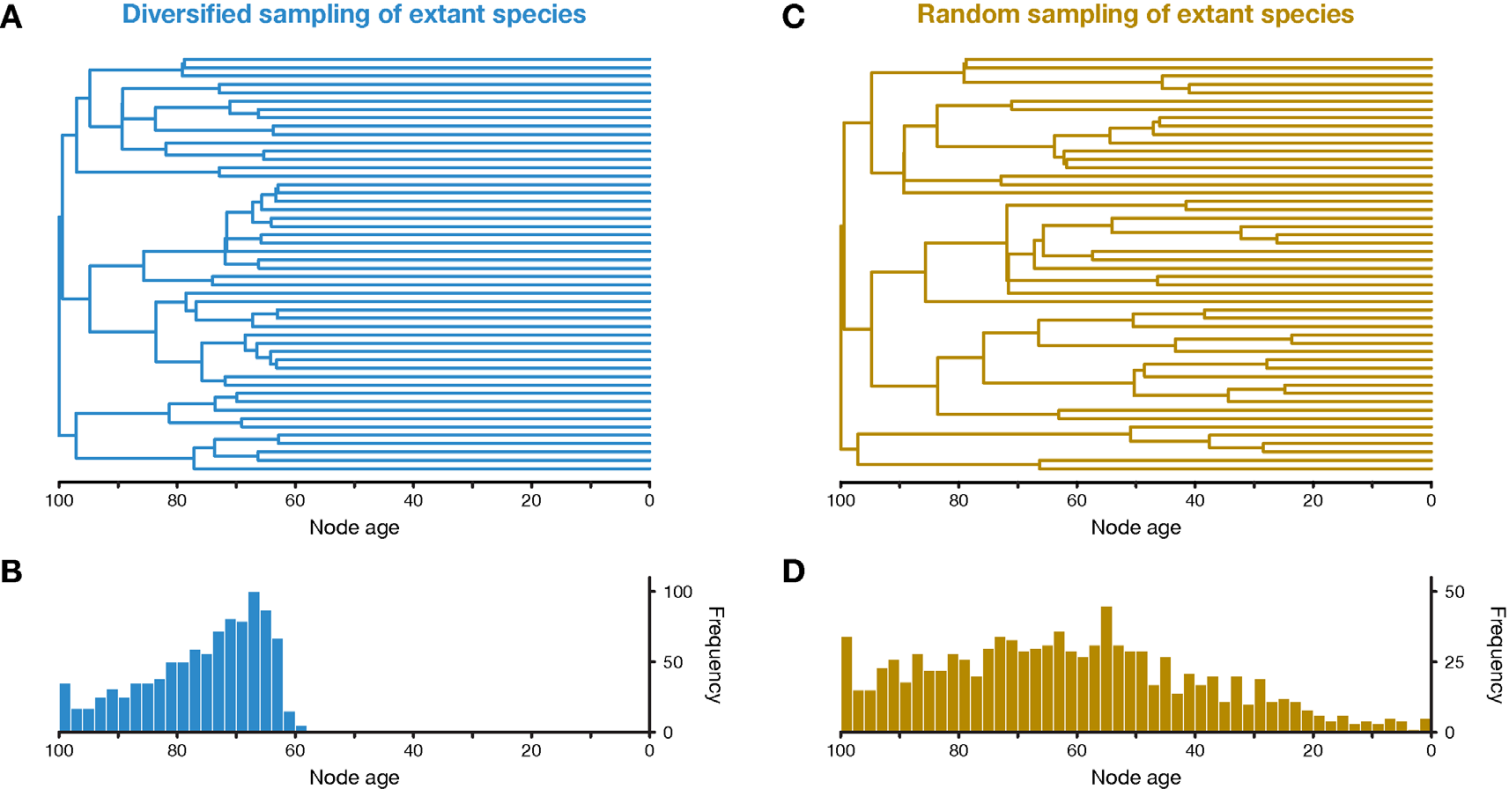
Simulation of phylogenetic trees. **(A)** One out of 20 diversified trees simulated for this study, after applying the diversified sampling scheme to sample 50 out of 4,000 to 5,000 extant species. **(B)** Distribution of node ages in all 20 diversified trees. **(C)** One out of 20 randomly sampled trees. **(D)** Distribution of node ages in all 20 randomly sampled trees.

To mimic the information content of empirical datasets in which fossils are assigned to extant clades, but no morphological data are available to infer interrelations, extinct branches were pruned from all simulated phylogenies and their fossil records were transferred to the ancestral branch in the reconstructed phylogeny from which they had diverged. Each internal branch thus represented the stem of an extant clade and the fossils assigned to the branch can be interpreted as the stem group of that clade. The ages of these fossils did not necessarily fall into the time period covered by the branch, but, as is the case for stem-group fossils, could postdate the origin of the crown group and thus the end of the stem branch. I then selected 50 extant species from each tree according to the strict diversified sampling scheme of Höhna et al. (2011), meaning that first, the time point in the phylogeny was identified at which 50 branches with extant descendants existed, and second, one of these descendants is sampled at random for each of these 50 branches. The tree was then reduced to the branches connecting these 50 species, the “diversified tree” (**Figure 1**). As a consequence of this sampling scheme, the diversified tree is guaranteed to include the 49 oldest divergences among extant species but none of the divergences that are younger than those 49. In addition to the diversified sampling scheme, I separately applied the random sampling scheme, sampling 50 extant species uniformly at random with the only requirement that at least one extant species was sampled from both sides of the root. For both the diversified tree and the randomly sampled tree, the fossil records of pruned branches were once again transferred to the corresponding ancestral branches remaining in the phylogeny. As in Matschiner et al. (2017), nucleotide sequences of a length of 3,000 base pairs (bp) were simulated along each tree according to the unrestricted empirical codon model of Kosiol et al. (2007) with a mean substitution rate set to 3 × 10-3^-3^ and a rate-variance parameter of 9 × 10-6^−6^.

### Divergence-Time Estimation With the FBD Model and Cladeage

For each of the 20 datasets simulated with the diversified sampling scheme and the 20 datasets generated with random sampling, I estimated divergence times among the 50 species with both the FBD model (Gavryushkina et al., 2014; Heath et al., 2014) and CladeAge (Matschiner et al., 2017). I performed these analyses either with the FBD model implementation in the SA package v.1.1.7 or the CladeAge implementation in the CA package v.1.3.0, both of which are add-ons for the software BEAST 2 (Bouckaert et al., 2019) (of which I used v.2.4.2). As starting trees in analyses with the FBD model, I prepared two modified versions of each diversified and randomly sampled tree in which either only the oldest fossil per branch or all fossils of each branch were inserted as extinct tips and connected to their respective branches *via* newly added branches. The topology of extant species was fixed to their true topology; however, as in Matschiner et al. (2017), this was done with “CladeConstraint” topology constraints (Gavryushkina et al., 2014) in the case of FBD analyses, so that fossils were allowed to attach either on the stem or in the crown of the clade to which they were assigned.

Using four different settings (“Set1” to “Set4”) in FBD analyses, the parameters of the FBD model implementation in BEAST 2, net diversification rate (λ−μ), turnover (μ/λ), and fossil-sampling proportion (ψ/(μ+ψ)) (Gavryushkina et al., 2014), were either fixed (Set1,3) to the true values used in simulations (0.06, 0.5, and 0.143; see above) or estimated (Set2–4; **Table 1**), and all three parameters were assumed constant throughout the tree. When these parameters were estimated, uniform priors were used as constraints for each of them, centered on the true values and with lower and upper boundaries corresponding to 50% and 150% of the true value, respectively. The probability of sampling extant species, *ρ*, was fixed to the true proportion of sampled species (thus, 50 divided by the number of extant species in the full simulated tree; a value between 0.01 and 0.0125). In most analyses with the FBD model (Set1–3), the fossil records were reduced to the oldest fossil per branch, but an additional set of analyses (Set4), in which diversification and sampling parameters were estimated, was also conducted with all fossils of each branch (**Table 1**). For computational reasons, the setting Set4 was only applied to datasets generated with diversified sampling of extant species.

**Table 1:** Settings, results, and run statistics for analyses of simulated datasets with the FBD model and CladeAge. The simulated datasets were either based on diversified or random sampling of extant species. Accuracy (percentage of estimates within 95% HPD interval), root-mean-square-deviation between true ages and mean node-age estimates (RMSD), iterations to stationary, time per iteration, and time to stationarity are averaged over the analyses of 20 simulated datasets.

Species sampling	Inference setting	Method	Diversification parameters	Fossil-sampling parameters	Fossils used	Accuracy	RMSD	Iterations to stationary	Time per iteration	Time to stationarity
Diversified	Set1	FBD	Fixed	Fixed	Oldest	78.5%	6.0	76.7M	0.643 ms	13.6 h
Diversified	Set2	FBD	Estimated	Estimated	Oldest	0.6%	55.8	101.1M	1.012 ms	25.4 h
Diversified	Set3	FBD	Estimated	Fixed	Oldest	5.5%	38.1	54.1M	0.967 ms	13.8 h
Diversified	Set4	FBD	Estimated	Estimated	All	89.4%	6.6	>1,200.0M	3.224 ms	>1,074.5 h
Diversified	Set5	CladeAge	Fixed	Fixed	Oldest	90.3%	4.6	49.2M	0.594 ms	8.5 h
Diversified	Set6	CladeAge	Estimated	Estimated	Oldest	91.0%	4.6	58.9M	0.551 ms	8.6 h
Random	Set1	FBD	Fixed	Fixed	Oldest	90.7%	4.8	80.8M	0.578 ms	12.9 h
Random	Set2	FBD	Estimated	Estimated	Oldest	2.9%	69.2	53.8M	0.825 ms	13.1 h
Random	Set3	FBD	Estimated	Fixed	Oldest	6.3%	52.1	57.2M	0.627 ms	9.9 h
Random	Set5	CladeAge	Fixed	Fixed	Oldest	91.6%	4.6	39.3M	0.550 ms	5.8 h
Random	Set6	CladeAge	Estimated	Estimated	Oldest	91.5%	4.7	35.2M	0.523 ms	5.0 h

In contrast to the FBD model, CladeAge does not assume that the diversification parameters of the tree-generating process are identical to those of the fossil-generating process; thus, these parameters need to be specified separately for the fossil-generating process. While they could also be specified differently for each clade, I here always used the same values for all clades. The fossil-generating process is parameterized as in the FBD model implementation in BEAST 2 with net diversification rate (λ−μ) and turnover (μ/λ), but instead of the fossil-sampling proportion (ψ/(μ+ψ)), the sampling rate (ψ) is used. Analogous to the analyses with the FBD model, I specified the three parameters either exactly according to their true values (Set5) or applied confidence intervals with lower and upper boundaries set to 50% to 150% of the true parameter values (Set6). The tree-generating process, on the other hand, was in all CladeAge analyses assumed to be the birth–death process (Gernhard, 2008) and uninformative uniform priors were used for the two parameters of the birth–death process, the net diversification rate (λ−μ; constrained to λ−μ∈[0,1,000]) and the turnover (μ/λ; constrained to μ/λ∈[0,1]). Conforming to the assumptions of CladeAge, fossil records were reduced to the oldest fossil per branch in all CladeAge analyses and fossils were reused for parental branches if these did not have a fossil record on their own (see **Figure 2A** in Matschiner et al., 2017).

**Figure 2:**
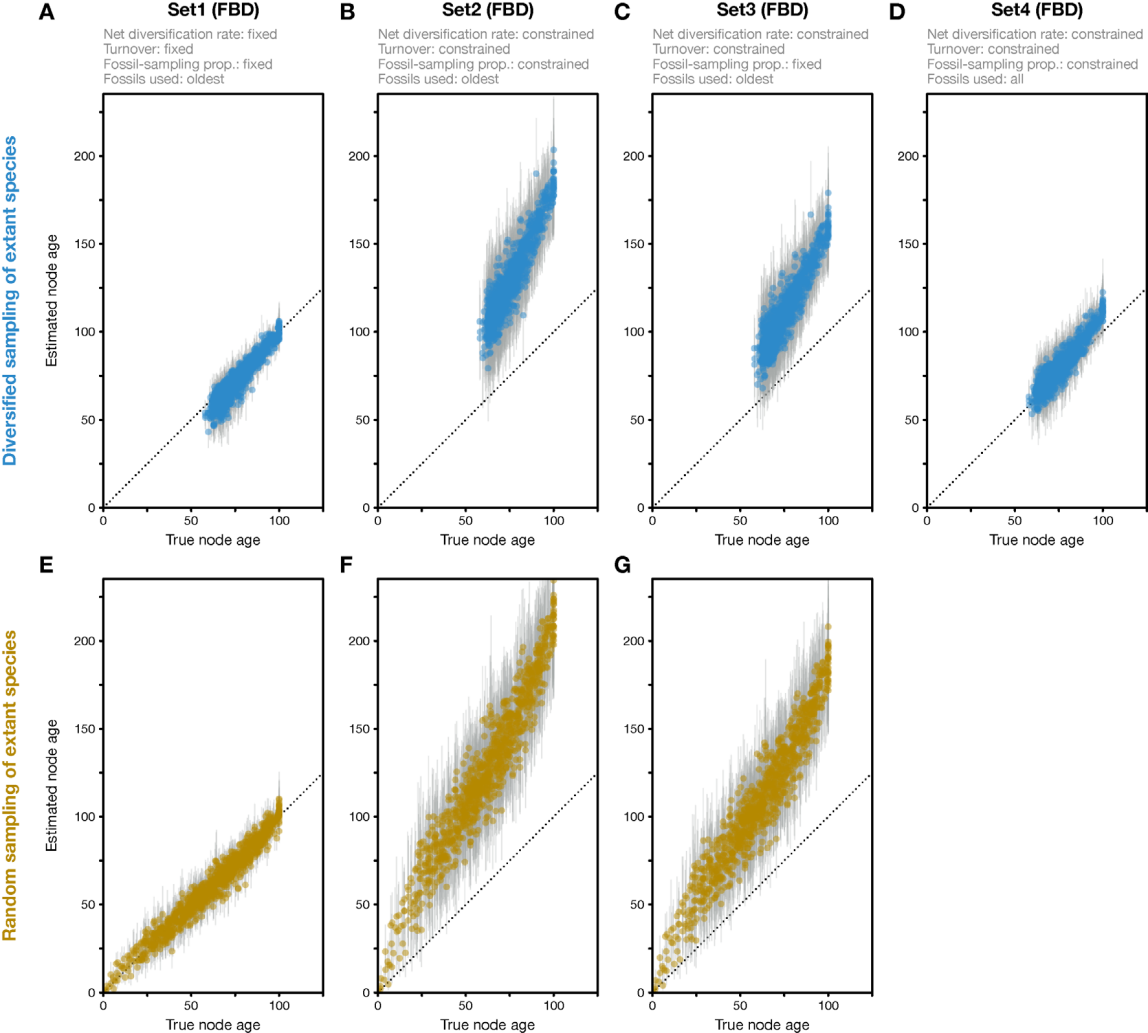
Divergence times estimated with the FBD model. **(A)** Comparison of true node ages and node ages estimated in FBD analyses of simulated diversified trees, using inference setting Set1 in which diversification and sampling parameters were fixed to their true values. The dotted line marks the diagonal. **(B)** Node-age estimates for FBD analyses with setting Set2 in which net diversification, turnover, and fossil-sampling proportion were estimated. **(C)** Node-age estimates for FBD analyses with setting Set3 in which net diversification and turnover were estimated but the fossil-sampling proportion was fixed to its true value. **(D)** As B but for FBD analyses with setting Set4 in which all fossils of each branch were used instead of reducing the fossil records to the oldest fossils per branch. **(E**–**G)** As A–C but for datasets generated based on random sampling of extant species. Analyses with all fossils were conducted only for diversified trees, not for randomly sampled trees.

In all analyses, sites of the sequence alignment were grouped into three different partitions according to codon position, and the reversible-jump-based substitution model of Bouckaert et al. (2013) was applied to each of them. Branch-rate variation was modeled with the uncorrelated clock model of Drummond et al. (2006). All analyses were set to use 100 million Markov-chain Monte Carlo iterations but were resumed after finishing if the chain had not reached stationarity. To assess stationarity, effective sample sizes of all model parameters (ESS) were calculated with the coda R package v.0.19 (Plummer et al., 2006) and considered sufficient if all of them were above 200. After reaching stationarity, the length of the burnin period was determined visually from trace plots generated with Tracer v.1.7.1 (Rambaut et al., 2018) and the minimum number of iterations required to reach stationarity post-burnin was calculated, again with the coda R package. Finally, the results of each set of 20 analyses that shared identical simulation (diversified or random sampling) and inference (Set1–Set6) settings were pooled before interpretation. The accuracy of divergence-time estimates was quantified as the proportion of 95% highest-posterior-density (HPD) intervals (across the 20 analyses) that included the true node age. Without model violations, an accuracy of 95% would be expected. All BEAST 2 analyses made use of the BEAGLE computing library v.4.1 (Ayres et al., 2012) and were carried out with three threads on dual eight-core Intel Xeon E5-2670 (Sandy Bridge-EP) CPUs running at 2.6 GHz.

## Results

### Simulations

The 20 simulated trees had on average 4,490.6 (standard deviation, sd: 226.0) extant species. After applying the diversified sampling scheme, all terminal branches were longer than 57.9–68.3 time units (mean across trees: 63.2; sd: 2.2) and all divergences were thus concentrated within the first 31.7–42.1 time units of the diversified tree (**Figures 1A, B**). This was reflected by the γ statistic of the constant-rates test of Pybus and Harvey (2000), which was highly negative for all 20 diversified trees (mean: −10.1; sd: 0.2). Qualitatively, the diversified trees appeared similar in shape to time-calibrated phylogenies of larger clades based on genomic datasets, such as the phylogeny of birds by Jarvis et al. (2014) or the phylogeny of spiny-rayed fishes by Alfaro et al. (2018). In contrast, random sampling of extant species produced trees with a wider distribution of node ages and shorter terminal branches (**Figures 1C, D**). The γ statistic was closer to zero but still negative for randomly-sampled trees (mean: −6.8; sd: 0.6), as expected due a decline in the “pulled speciation rate” near the present in cases of incomplete sampling of extant species (Louca and Pennell, 2019). The simulated fossil records included between 672 and 800 fossils (mean across trees: 737.4; sd: 37.8) that attached to 52–59 branches of the diversified trees or 62–72 branches of the randomly-sampled trees. This increased number of branches with fossils in randomly-sampled trees can be explained by the smaller number of very short branches compared to diversified trees (see **Figures 1A, C**). The sequence alignments of 3,000 bp simulated for diversified trees contained between 2,741 and 2,890 (mean: 2,824.8; sd: 34.6) variable sites, out of which 1,737–2,141 (mean: 1,962.4; sd: 94.0) sites were parsimony-informative. For randomly-sampled trees, between 2,526 and 2,857 (mean: 2,722.1; sd: 73.9) sites were variable, including 1,579–2,183 (mean: 1,913.6; sd: 1,35.1) parsimony-informative sites.

### Divergence-Time Estimation With the FBD Model and Cladeage

**Figure 3:**
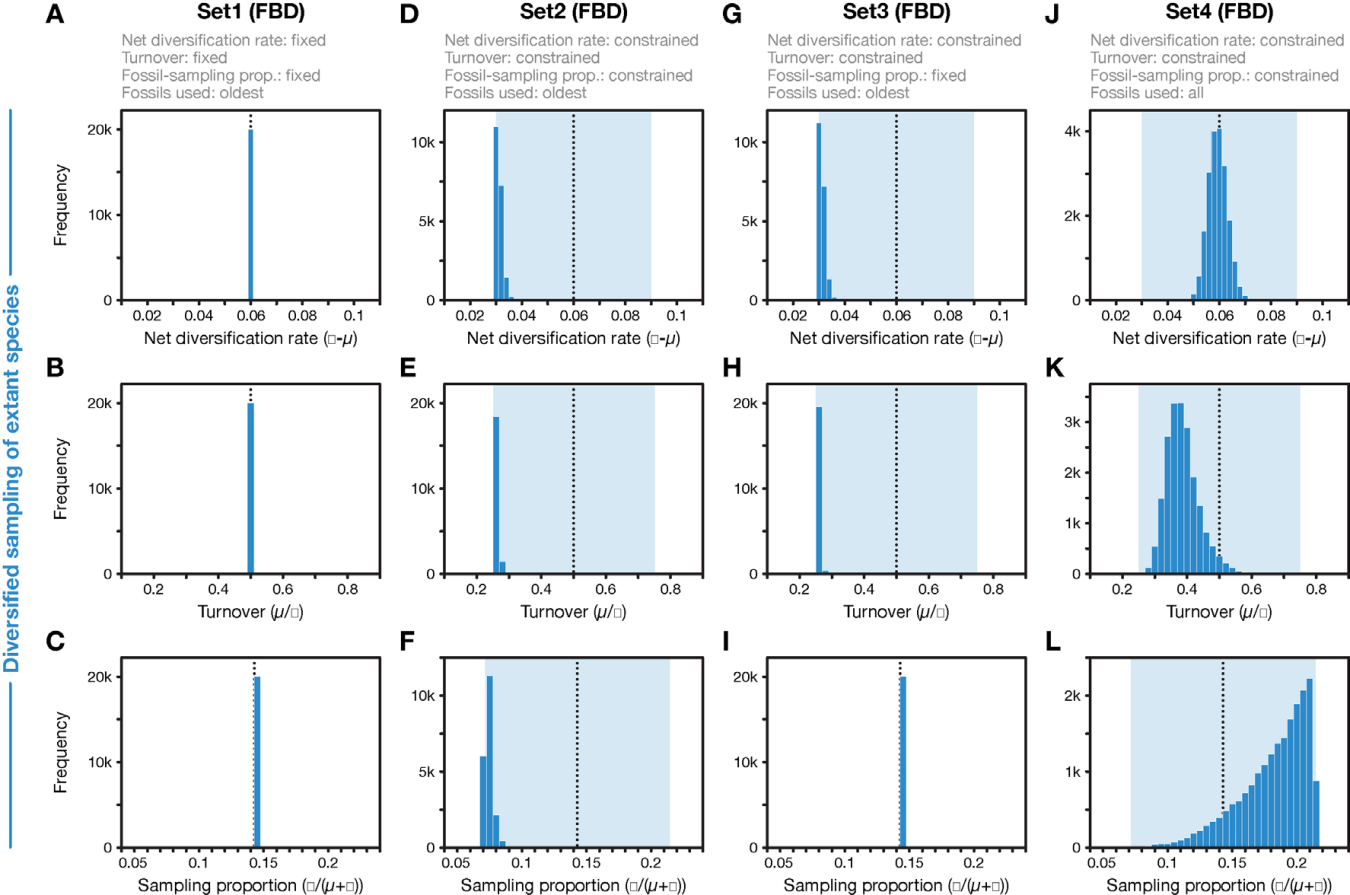
Parameter values for net diversification rate, turnover, and fossil-sampling proportion, estimated with the FBD model. **(A**–**C)** In FBD analyses with inference setting Set1, diversification and sampling parameters were fixed to their true values, indicated with vertical dotted black lines. **(D**–**F)** In FBD analyses with setting Set2, net diversification, turnover, and fossil-sampling proportion were estimated with uniform prior intervals shown in light blue; the histograms show the posterior distributions. **(G**–**I)** As D–F but for FBD analyses with setting Set3 in which the fossil-sampling proportion was fixed to its true value. **(J**–**L)** As D–F but for FBD analyses with setting Set4 in which all fossils of each branch were used instead of reducing the fossil records to the oldest fossils per branch. All results shown here were obtained with the 20 simulated diversified trees; those obtained with random sampling of extant species were nearly identical (for **A**–**I)**.

When all diversification and fossil-sampling parameters were fixed to the true values used in simulations (Set1), age estimates obtained with the FBD model were relatively accurate despite the model violations of diversified sampling and the reduction of the fossil record to the oldest fossils per branch. With these settings, 78.5% of the 95% HPD intervals contained the true node age (**Table 1**), and the mean age estimates appeared close to the true ages (root-mean-square deviation, RMSD: 6.0 time units) (**Figures 2A**, **3A-C**). However, when diversification and fossil-sampling parameters were estimated instead of fixed to the true values (Set2), almost all ages were substantially overestimated (RMSD: 55.8). In this case, only 0.6% of the 95% HPD intervals included the true node age and every single mean node-age estimate was older than the true age (**Figure 2B**). The low accuracy of node ages was reflected by the estimates of the net diversification rate, the turnover, and the fossil-sampling proportion, all of which appeared at the lower boundaries of the uniform prior intervals used as constraints (**Figures 3D–F**). Fixing only the fossil-sampling proportion to the true value while estimating the diversification parameters (Set3) led to a moderate improvement in the node-age estimates, resulting in an accuracy of 5.5% and slightly lower mean node ages (RMSD: 38.1) (**Figure 2C**). The estimates of the net diversification rate and the turnover, however, remained near the lower prior boundary (**Figures 3G–I**). In contrast, the use of all fossils instead of only the oldest per branch (Set4) resulted in a much better accuracy of node-age estimates, namely 89.2% (RMSD: 6.6) (**Figure 2D**). In this set of analyses, estimates of the net diversification rate were centered close to the true value (λ−μ = 0.06) (**Figure 3J**), and while the turnover and the fossil-sampling proportion appeared to be under- and overestimated, respectively, the posterior distributions of these estimates included the true values (**Figures 3K, L**).

The computational requirements of FBD analyses with all fossils, however, were far larger than those of all other analyses. Whereas the FBD analyses with only the oldest fossils per branch of the diversified tree required between 54.1 and 76.7 million MCMC iterations to stationarity and these completed within 13.6 to 25.4 h, all but one of the 20 FBD analyses with all fossils had not reached stationarity even after 1.2 billion MCMC iterations that took 1,074.5 h (45 days) (**Table 1**). The lowest ESS value after this number of iterations was 24.2, suggesting that around 10 billion iterations and a run time of around a year might be necessary to reach ESS values greater than 200 for all parameters in all analyses of Set4.

The analyses of datasets generated with random sampling of extant taxa produced results similar to those based on diversified sampling (**Figures 2E–G**). When all diversification and fossil-sampling parameters were fixed to their true values, node ages estimated with the FBD model were largely accurate (**Figure 2E**), with 90.7% of the 95% HPD intervals containing the true node age (**Table 1**). In contrast, allowing all model parameters, or only the diversification parameters, to be estimated led to a degree of node age overestimation that was even larger than in the results based on diversified sampling of extant species (RMSD: 69.2 and 52.1 without and with fixing the fossil-sampling proportion, respectively) (**Figures 2F, G**). Due to their great computational requirements, I did not conduct FBD analyses with all fossils for the randomly sampled trees. However, as these FBD analyses with all fossils of randomly sampled trees would not suffer from model violations due to selective sampling of species or fossils, I assume that they would provide accurate estimates of node ages and model parameters.

**Figure 4:**
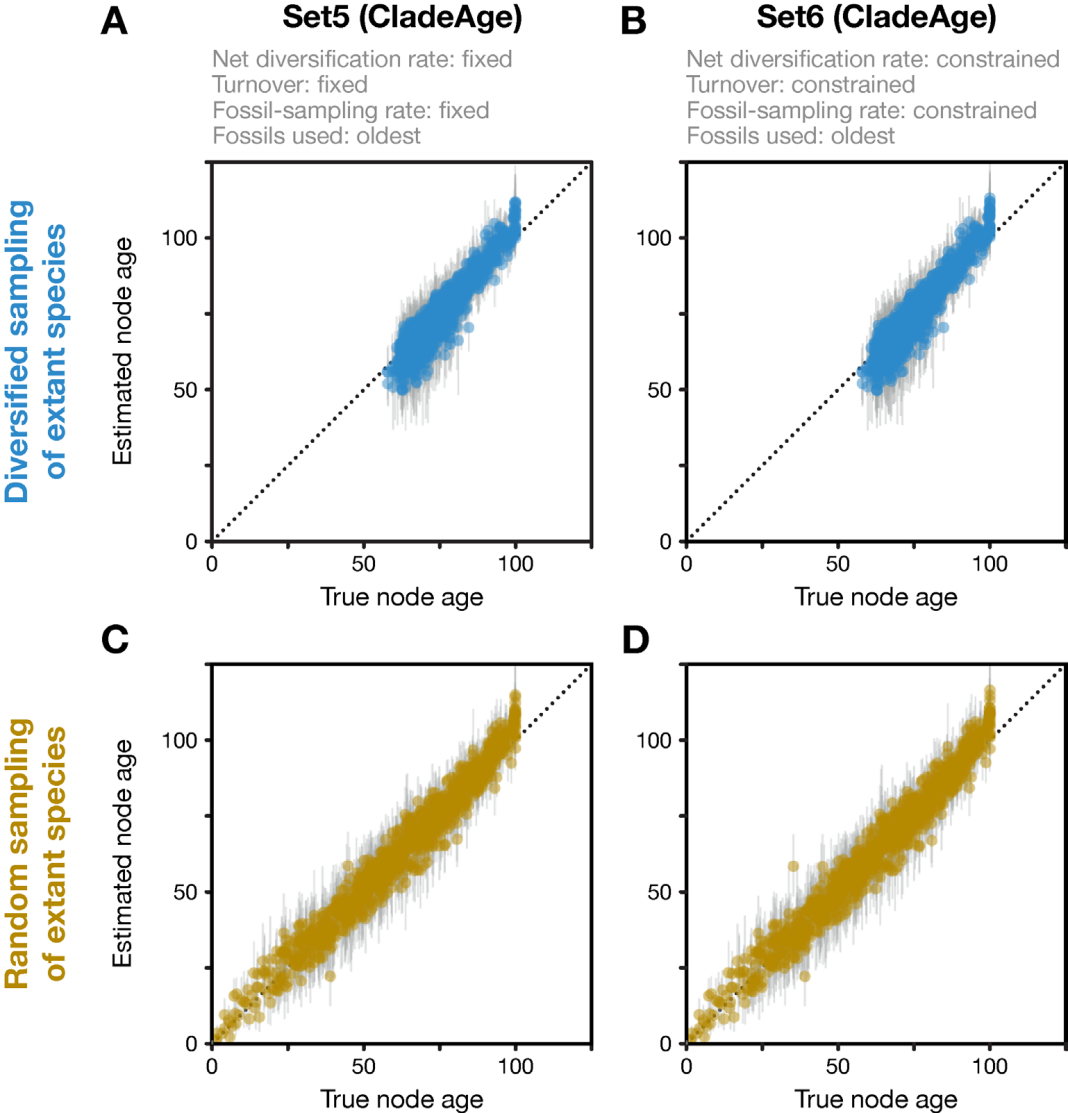
Divergence times estimated with CladeAge. Comparison of true node ages and estimated node ages for the 20 diversified trees **(A**, **B)** and the 20 randomly sampled trees **(C**, **D)**. In the inference, diversification and sampling parameters were either specified exactly **(A**, **C)** or as confidence intervals **(B**, **D)**. The dotted lines mark the diagonals.

Analyses with CladeAge were not affected by the reduction of the fossil record to the oldest fossils as this reduction is already expected by CladeAge. Regardless of whether datasets had been generated with diversified or random sampling of extant species and whether the diversification and sampling parameters were specified exactly (Set5) or considered uncertain within intervals ranging from 50% to 150% of the true values (Set6), the accuracy of node-age estimates was above 90% (90.3–91.6%) and mean node-age estimates were close to the true values (RMSD: 4.6–4.7) (**Figure 4**). Run times with CladeAge were comparatively short; on average, between 35.2 and 58.9 million iterations were required to reach stationarity and these numbers of iterations completed within 5.0 to 8.6 h (**Table 1**).

## Discussion

My analyses of simulated data show that the FBD model can produce highly inflated age estimates when sampling of species and fossils is not complete or random but selective. Because of their great computational demand, I did not perform analyses in which species were sampled randomly (or completely) and all fossils were used; however, based on the results of previous studies (Gavryushkina et al., 2014; Matschiner et al., 2017), I assume that these analyses would have resulted in high accuracy close to 95%. This would mean that the decrease in accuracy of node-age estimates (to 89.2%) was minor when only the diversifed sampling of extant species was applied but the entire fossil record was used for calibration in analyses with setting Set4. A far more dramatic decrease in accuracy (down to 0.6%), along with substantial overestimation of node ages, resulted from the reduction of the fossil record to the oldest fossils per branch in analyses with setting Set2. The comparison of the results obtained with settings Set2 and Set4 thus allows us to disentangle the effects of the two types of model violation and interpret how they may have led to the observed node-age overestimation.

First, the underestimated turnover observed in the FBD analyses with setting Set4 may be explained by the bottom-heavy shape of the diversified trees (**Figure 1**), a pattern that is opposite to that expected from high turnover, a concentration of divergences among extant species near the present. Underestimates of turnover, in turn, imply that the number of extinct branches is also underestimated, which could be responsible for overestimation of the fossil-sampling proportion in the analyses with setting Set4 based on unreduced fossil records. In the FBD analyses with setting Set2, however, the reduction of fossil records to the oldest fossils per branch may have counteracted the overestimation of the fossil-sampling proportion, leading even to strong underestimation of this proportion. The more accurate estimates of turnover in analyses of Set4 with all fossils, compared to those of Set2 with only the oldest fossils, are likely explained by the large number of additional extinct branches in the phylogenies of Set4 that support a higher extinction rate (μ) and thus a higher turnover (μ/λ).

As the comparison of results obtained with settings Set4 and Set2 shows, it is the selective sampling of the oldest fossils per branch that is responsible for most of the overestimation of node ages in analyses with setting Set2. Thus, it might be surprising that by fixing the sampling proportion to its true value in analyses with setting Set3, only moderate improvements in age estimates are gained. How can the selective sampling of fossils impact age estimates if not through the sampling proportion? The answer probably lies in the indirect relationship between fossil-sampling proportion and fossil-sampling rate ψ, which is influenced by the extinction rate μ, as the fossil-sampling proportion is ψ/(μ+ψ). If, as is roughly the case in the analyses with settings Set2 and Set3, both the net diversification rate (λ−μ) and the turnover (μ/λ) are estimated as half of their true values (0.06 and 0.5, respectively), this means that the extinction rate is implicitly estimated as μ = 0.01 (and the speciation rate is implicitly estimated as λ = 0.04). The estimated extinction rate is thus only a sixth of the true value used in the simulations (μ = 0.06; see above). With an estimated extinction rate μ = 0.01 and the fossil-sampling proportion fixed at ψ/(μ+ψ) = 0.143, the fossil-sampling rate is ψ = 0.00167, also a sixth of the true value used in the simulations. Thus, despite fixing the fossil-sampling proportion in setting Set3, the fossil-sampling rate ψ that is implicit in the model remains substantially underestimated. With underestimated fossil-sampling rates, the expected waiting times between clade ages and their first fossil records increase, and as a result, older trees become more probable under the FBD model, leading to the observed overestimated node ages. However, the underestimation of the fossil-sampling rate with setting Set3 is not as severe as in the analyses with setting Set2 (with the fossil-sampling proportion estimated around ψ/(μ+ψ) = 0.071 in those analyses, the implicitly estimated fossil-sampling rate is ψ = 0.00077), which likely explains the modest improvements in node-age estimates between analyses with setting Set2 and those with Set3. In the analyses with setting Set1, on the other hand, fixing of all three explicit model parameters net diversification rate (λ−μ), turnover (μ/λ), and fossil-sampling proportion (ψ/(μ+ψ)) also fixes the implicit model parameters speciation rate (λ), extinction rate (μ), and fossil-sampling rate (ψ) to the true values used in the simulations, explaining the largely accurate age inference in those analyses.

The issues highlighted by my analyses of simulated data suggest that the application of the FBD model to larger empirical datasets may often be problematic. To investigate divergence times of species-rich clades with large fossil records like placental mammals, birds, or teleost fishes with the FBD model, researchers would need to decide between the options of complete, random, or selective fossil sampling, all of which are not ideal. Complete sampling of the fossil records of these clades would entail the use of thousands of fossils, but as my analyses with setting Set4 showed, even hundreds of fossils, in combination with rather small molecular datasets, require prohibitive run times of months or years. Whereas future improvements to FBD implementations may shorten these run times to some extent, it is questionable whether analyses with thousands of fossils and large molecular datasets will ever become computationally feasible (note, however, that by not using molecular data, the FBD implementation of Didier and Laurin (2018) allows rapid inference with larger numbers of fossils). On the other hand, random sampling of the fossil record may provide feasible run times and largely unbiased age estimates, but as the random sampling scheme may often exclude the oldest fossils of clades, the estimated ages of these clades may sometimes be younger than their oldest fossils if the molecular data do not permit sufficiently precise estimates. In contrast, as shown by my FBD analyses with settings Set2 and Set3, the sampling scheme in which only the oldest fossils per clade are used results in strongly overestimated node ages when the values of diversification and fossil-sampling parameters are not known exactly.

The results obtained with setting Set4 further suggest that even when all fossils are used in FBD analyses, diversified sampling of extant species leads to moderately inaccurate estimates of node ages, turnover, and fossil-sampling proportion. When the empirical sampling of extant species is in fact strictly according to the diversified sampling scheme, this issue could be solved with FBD model implementations that explicitly account for this scheme. An FBD model implementation with this feature is available in the program MrBayes (Zhang et al., 2016) but is so far missing from BEAST 2 (Bouckaert et al., 2019). However, even though many empirical datasets may be designed to include a diverse set of species, they are unlikely to follow the diversified sampling scheme strictly (Höhna, 2014). The reason for this is that the strict diversified sampling scheme expects all nodes up to a certain age, but no nodes with younger ages, to be sampled, but this age information is not usually available to researchers prior to the analysis (Höhna et al., 2011). As a result, the use of FBD implementations that account for strict diversified sampling may result in node-age estimates that are biased in the opposite direction, towards underestimation, when empirical datasets are compiled with a sampling scheme that is intermediate between diversified and random sampling (Harrington and Reeder, 2017). A “semi-diversified” sampling scheme that could often be more appropriate for empirical datasets has been described and used for simulations by Colombo et al. (2015), but is not available for inference. In cases where each sampled species represents a clade with known species richness and no clades are missing from the phylogeny, the “empirical taxon sampling” scheme, which is available in RevBayes and accounts for varying fossil-sampling proportions across clades, might allow unbiased inference with RevBayes’ FBD implementation. Testing this assumption with simulations should be the focus of a future study.

In contrast to the FBD model, CladeAge produced largely accurate node-age estimates after short run times, regardless of whether datasets had been generated with diversified or random sampling of extant taxa and regardless of whether diversification and sampling parameters were fixed or constrained within intervals. This difference between the models likely has two reasons: First, CladeAge explicitly assumes that only the oldest fossils per clade are used for calibration whereas the reduction of fossil records to the oldest fossils violates the FBD model. Second, whereas the FBD model assumes that the same diversification and sampling parameters apply to the fossil-generating process and the tree-generating process, this is not the case for CladeAge and thus, this method may be better able to buffer the model violation of diversified sampling by adjusting the diversification parameters of the tree-generating process without affecting the way in which fossils calibrate the tree. However, unlike the FBD model, CladeAge is unable to estimate the parameters of the fossil-generating process from the data, and these parameters therefore need to be specified by the user. While rough estimates of these parameters may be available from published literature (see, e.g., references in Supplementary Table 1 of Matschiner et al., 2017), separate analyses of clade-specific fossil records may in many cases be required to obtain these estimates, for example with the programs PyRate (Silvestro et al., 2014) TRiPS (Starrfelt and Liow, 2016), or Diversification (Didier et al., 2017). To obtain accurate divergence-time estimates, users should thus ensure that either their dataset conforms to the expectations of the FBD model—then this model will allow accurate estimation—or that estimates for diversification and sampling parameters are available *a priori*—then CladeAge can be used for the inference.

## Data Availability Statement

Code to reproduce this study can be found on GitHub: https://github.com/mmatschiner/fbd_test. 

## Author Contributions

MM conceived and designed the study, performed simulations, analyzed results, prepared figures, and wrote the manuscript.

## Funding

This research was funded by the Norwegian Research Council (FRIPRO 275869).

## Conflict of Interest

The author declares that the research was conducted in the absence of any commercial or financial relationships that could be construed as a potential conflict of interest.
